# A MICROLONGITUDINAL ASSESSMENT OF SUBJECTIVE AGE AND MENTAL HEALTH DURING REHABILITATION

**DOI:** 10.1093/geroni/igz038.199

**Published:** 2019-11-08

**Authors:** Amit Shrira, Yuval Palgi, Noemi Heyman, Oleg Zaslavsky, Ehud Bodner

**Affiliations:** 1 Bar Ilan University, Ramat Gan, Israel, Israel; 2 University of Haifa, Haifa, Hefa, Israel; 3 Shoham Geriatric Center, Pardes Hanna-Karkur, HaZafon, Israel; 4 University of Washington, Seattle, Washington, United States; 5 Bar-Ilan University, Ramat Gan, HaMerkaz, Israel

## Abstract

Recent ecological momentary assessments focused on the concomitants of daily views on aging among community-dwelling participants, yet clinical samples are underexplored. Hence, this study examined the relationships between views on aging and daily mental health during rehabilitation following osteoporotic fractures and cerebrovascular events. Measures of daily subjective age, psychological distress, and mental health were assessed among 132 older adult patients (mean age=77.9, SD=7.5, 65.9% women). Multilevel models showed that on days patients felt younger, they reported lower psychological distress and higher mental health. Time lagged analyses further showed reciprocal effects between subjective age and mental health. Finally, the subjective age-mental health covariance was stronger among patients high on age awareness. The suddenness and brutality of acute medical events highlight subjective age as an important factor in patients’ wellbeing, especially among those more attentive to their age. These findings suggest that practitioners should consider interventions focused on patients’ age identity.

